# Point Protection with Transfluthrin against *Musca domestica* L. in a Semi-Field Enclosure

**DOI:** 10.3390/insects15040277

**Published:** 2024-04-16

**Authors:** Robert L. Aldridge, Alexandra A. Pagac, Edmund J. Norris, Daniel L. Kline, Christopher J. Geden, Kenneth J. Linthicum

**Affiliations:** Center for Medical, Agricultural, and Veterinary Entomology (CMAVE), U.S. Department of Agriculture-Agricultural Research Service (USDA-ARS), Gainesville, FL 32608, USA; alexandra.pagac@usda.gov (A.A.P.); edmund.norris@usda.gov (E.J.N.); dan.kline@usda.gov (D.L.K.); chris.geden@usda.gov (C.J.G.); kenneth.linthicum@usda.gov (K.J.L.)

**Keywords:** spatial repellent, point protection, filth fly, volatile pyrethroid

## Abstract

**Simple Summary:**

House flies (*Musca domestica*) are a significant nuisance and vector species. Currently, few technologies are available to adequately control their populations, and insecticide resistance threatens the efficacy of some approaches currently utilized. Transfluthrin is a promising volatile pyrethroid that has been successful in controlling mosquitoes and other pest insects, offering the potential as an alternative or future chemical control tool for the abatement of diverse arthropod pests. For this study, we tested whether transfluthrin could prevent fly capture at an attractant source compared to the control when placed on the exterior of the attractant device. Our results indicate that transfluthrin significantly reduced fly capture for both a pyrethroid-susceptible and pyrethroid-resistant strain of house fly compared to the untreated controls. These results indicate the potential of using transfluthrin in future integrated pest management programs for the control of house flies.

**Abstract:**

House flies are notoriously difficult to control, owing to their tendency to live in close relationships with humans and their livestock, and their rapid development of resistance to chemical controls. With this in mind, we explored an alternative chemical control, a spatial repellent to deter *Musca domestica* L. from points we wanted to protect (i.e., a baited trap). Our results demonstrated that the synthetic spatial repellent, transfluthrin, is effective in preventing *M. domestica* adults from entering protected traps for both a susceptible strain (CAR21) and a field-acquired permethrin-resistant strain (WHF; 24 h LD_50_ resistance ratio of 150), comprising 22% and 28% of the total number of flies collected, respectively. These results are promising and demonstrate that transfluthrin can be an effective spatial repellent to protect points of interest where needed.

## 1. Introduction

House flies, *Musca domestica* L. (Muscidae, Diptera), are vectors of a number of pathogens that can be transmitted to humans and animals alike [1,2]. They are commonly called filth flies because they feed on filth as immatures and adults, and their contact with filth combined with their synanthropic behavior results in their propensity to vector pathogens [3,4]. Not all diseases associated with house fly-transmitted pathogens can be treated, so the next best option is to control the population that vectors the pathogen(s). There are several viable options that can control the vector population, but no option works 100% of the time due to complicating factors such as insecticide resistance or habitat. Therefore, specialists have developed integrated pest management (IPM) that combines multiple control strategies to more effectively control pest and vector populations [5].

Because insecticide resistance and product suspension/termination diminish the options for fly management with insecticides, novel approaches and adaptations for control must be explored [6]. One option is that of spatial repellents. Prior work on spatial repellents, including flunothrin, metofluthrin, and transfluthrin have shown promise in controlling mosquito vectors and some filth fly vectors. For example, Britch et al. [7] demonstrated that selected mosquito species could be repelled from entering treated enclosures. House flies were significantly repelled from pyrethroid-treated (including transfluthrin) tiles in Scrivener et al. [8]. Notably, Morrison et al. [9] demonstrated that transfluthrin significantly lowered the incidence of adverse epidemiological outcomes in an area where numerous mosquito-borne viruses were endemic (Iquitos, Peru).

Here, we investigated the use of a short-chain multi-halogenated benzyl pyrethroid, transfluthrin, as a spatial repellent to protect a point from incursion by two distinct strains of *M. domestica* [10]. As these strains had not been previously characterized for insecticide susceptibility, we evaluated the populations against each other through a contact exposure (topical application) dose response bioassay with a non-volatile pyrethroid, permethrin, demonstrating one fly strain to be highly resistant to pyrethroids. Then, we evaluated the degree to which transfluthrin could protect a point source (i.e., baited trap) in an outdoor semi-field enclosure after release of each of these two fly strains.

## 2. Materials and Methods

### 2.1. Rearing

The CAR21 susceptible strain of *M. domestica* (susceptibility confirmed by Baker et al. [11]) and WHF strain of *M. domestica* used in this study were reared at the USDA-ARS Center for Medical, Agricultural, and Veterinary Entomology (CMAVE), using the protocols outlined in Geden et al. [12]. The “wild” house flies (WHF) were originally collected from dairy farms in Florida, Minnesota, California, and Nebraska in 2014–2015. The four wild colonies were maintained separately until they were combined in 2017 [13], from which the flies have been reared without permethrin exposure. The susceptible strain of house flies (CAR21) was obtained from the Carolina Biological Supply (Burlington, NC, USA) in 2021, and has been reared without insecticide exposure since.

Flies used in the tests were of mixed sex, 2–4 days old, and were allowed to feed on sucrose and water ad libitum prior to bioassay treatment. Flies used for semi-field bioassays were restricted from feeding on sucrose for ca. 16 h prior to release. Both strains of flies were reared using the same methods, and all materials related to either were always kept separate to prevent the two strains from mixing.

Adult house flies were kept in screened metal cages (38 cm× 38 cm× 45 cm; 6 × 8 strand mesh/cm) and provided with food and water ad libitum. Flies were maintained at 26 ± 2 °C, 60 ± 5% RH, and a 12:12 h light/dark photoperiod. The adult house fly diet consisted of an 8:8:1 ratio of non-fat milk powder, granulated sugar, and powdered egg. Water was provided in a 1.9 L plastic bucket with expanded polystyrene foam packing “peanuts” placed on the surface of the water to act as a perch for the house flies. Colony cages were set with approximately 6000 puparia, 6 dishes containing 60 cm^3^ of fly diet, and a water bucket.

### 2.2. Permethrin Susceptibility Bioassay

Both strains (WHF and CAR21) were evaluated for susceptibility to permethrin. Female flies, 2–4 days old, were briefly anesthetized with CO_2_ and treated topically with 0.5 µL of technical permethrin diluted in acetone over a range of 2× dilutions, with acetone acting as a control. Concentrations of 0.005, 0.01, 0.02, 0.04, and 0.08 mg/mL (equivalent to 2.5–40 ng/fly) were applied to the CAR21 strain, and 0.64, 1.28, 2.56, 5.12, and 10.24 mg/mL (equivalent to 320–5120 ng/fly) were applied to the WHF strain. Flies were treated in groups of 20 females per dose on four separate occasions (4 replicates), with different batches of flies (80 flies per dose total) using 5 doses that resulted in mortality in the range of 5–95%. Treated flies were transferred to 162 mL cups with screen lids, provided with 10% sucrose on cotton balls, and assessed for mortality after 24 h.

### 2.3. Semi-Field Experimental Design

The goal of the experiment was to determine whether transfluthrin could protect, by spatial repellency, a point (i.e., a trap) from incursion by *M. domestica*. The experiments took place between June and August 2023. Weather conditions for this period, as reported by the weather station located at the regional airport approximately 9.92 km away, ranged between a low of 20.6 °C and a high of 35.6 °C. Humidity remained between 42% and 100% for the course of the experiment. If there was inclement weather at the time of release (i.e., rain, sustained heavy winds [>30 kph], or lightning), the release was either cancelled or postponed until conditions were more favorable for release.

Based on the average individual weight of the pupae, an estimated 3300 pupae were placed in a polystyrene bowl in a 47.5 cm× 47.5 cm× 47.5 cm BugDorm^®^ cage (Megaview Science Co., Ltd., Taichung, Taiwan) with a dish containing 60 cm^3^ of granulated sugar and a separate dish of water saturated cotton. Puparia were held for fly emergence and released in cages when they were 2–4 days old. Sugar was removed from the cages at the end of the day preceding a test to ensure their avidity at the time of testing. Based on the historical emergence rate data (ca. 10% mortality) for these strains, this method ensured that each cage held approximately 3000 flies.

**Figure 1:**
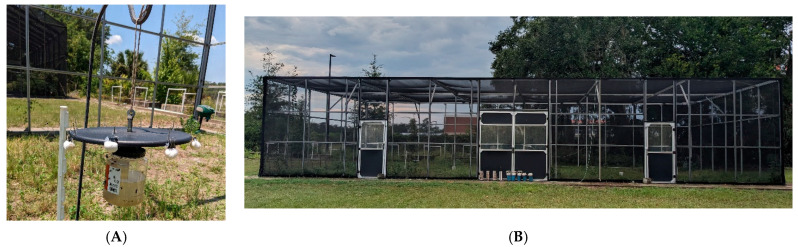
(**A**) Image of the modified Captivator trap collection container with cotton ball sachets. (**B**) Image of the semi-field enclosure utilized for this experiment; flies were released in this enclosure and collected in the modified Captivator traps with either control (water) or transfluthrin-treated cotton ball sachets.

The experiment was replicated for both the CAR21 and WHF strains. One strain (ca. 3000 flies) was released from its cage into a semi-field enclosure (9.4 m wide × 18.3 m long × 3.7 m high; 5 × 7 strand mesh/cm) located at CMAVE (Figure 1B). Approximately 90 min after release, 2 modified Captivator^®^ traps (Farnam Co., P.O. Box 34820, Phoenix, AZ, 85067, USA) containing liquid bait (Starbar^®^ Fly Trap Attractant, Central Life Sciences, Schaumburg, IL, USA), (Figure 1A) were placed in the enclosure parallel to the longest side, running north–south. Each trap was placed 2.3 m from the short side(s), at the midpoint between the long parallel sides, and 13.7 m apart from each other. Traps were suspended from shepherd hooks so that the base of the trap was 30–45 cm above the ground. Water containers (1.9 L) with styrofoam packing peanuts were placed along the same midline, but 4.6 m from each trap. The release site for the flies was the middle of the enclosure, 6.9 m from either trap. Packets of the attractant, composed of trimethylamine chloride, indole, z-9-tricocene, and putrescent egg solids [14] were mixed with 946 mL of water per product instructions immediately before trap placement. Traps were left in place (set at noon) and collected after 24 h.

Following collection, house flies were hand counted from each trap, which was either untreated or treated with transfluthrin (see below). Treated and control trap positions within the enclosure were alternated between replications to mitigate positional bias. Releases were performed for a total of 12 releases, 6 releases per strain.

#### Transfluthrin and Control Traps

A CDC light trap lid (35.6 cm dia. Bioquip products; Rancho Domingo, CA, USA) was modified with eight 1/8 inch (3.175 mm equivalent) aluminum rivets (with mandril retained) placed every 11–13 cm along its perimeter. A metal paper binder clip (32 mm wide) holding a sachet made from a 100–144 cm^2^ square of fine nylon mesh wrapped around a large cotton ball was hung from the mandril of each rivet. The sachet was treated either with tap water for the controls or 0.125 g AI of formulated transfluthrin (Bayothrin 200 EC; Bayer, Research Triangle, NC, USA) diluted in water. Eight sachets of formulated transfluthrin were placed on the lid, for a total of 1 g of active ingredient. An amount of 1 g was used as it was equivalent to prior experiments that targeted mosquito repellency utilizing 1 g/m^2^ of HESCO or camouflage netting material [7,15]. Control sachets were replaced after every deployment, and transfluthrin sachets were used two times but rotated between traps.

Captivator fly traps were altered by attaching a modified CDC light trap lid via a 5 cm steel bolt, that was molded into the Captivator trap lid first using heat to deform the soft plastic, and then secured using epoxy steel putty. The CDC trap lid was then kept in place by a 2.54 cm nut modified to be hung from a shepherd’s hook with a tan #550 paracord (Figure 1A).

### 2.4. Statistical Analysis and Data Visualization

The topical bioassay data were subjected to probit analysis using the Probit Procedure of the Statistical Analysis System version 9.4 (SAS Institute, version 9.2, Cary, NC, USA). Control mortality was low (5% or less) and not corrected using Abbott’s correction. Fly trap collections were recorded in Excel and analyzed using R (version 4.2.1 [2022-06-23 ucrt]) and the R studio GUI (version 1.2.1335). Collected *M. domestica* values were separated by strain, and both treatment and location variables were fitted to a generalized linear model (GLM), with a negative binomial error structure specified and a log link function, using the MASS package. Figures were generated through R and RStudio using the ggplot2 package.

## 3. Results

### 3.1. Permethrin Susceptibility Bioassay

House fly permethrin susceptibility bioassays were conducted for this experiment. The results demonstrate that the WHF strain is resistant to permethrin compared to the susceptible CAR21 strain with resistance ratios (RR) of 149.6 for the LD_50_ and 328.2 for the LD_90_, as shown in Table 1. Probit models were analyzed separately using Pearson’s goodness of fit test for the WHF strain (Χ^2^ = 6.163; df = 3; *p* = 0.104) and the CAR21 strain (Χ^2^ = 1.46; df = 3; *p* = 0.223).

### 3.2. Semi-Field with “WHF” Permethrin Resistant Strain

**Figure 2:**
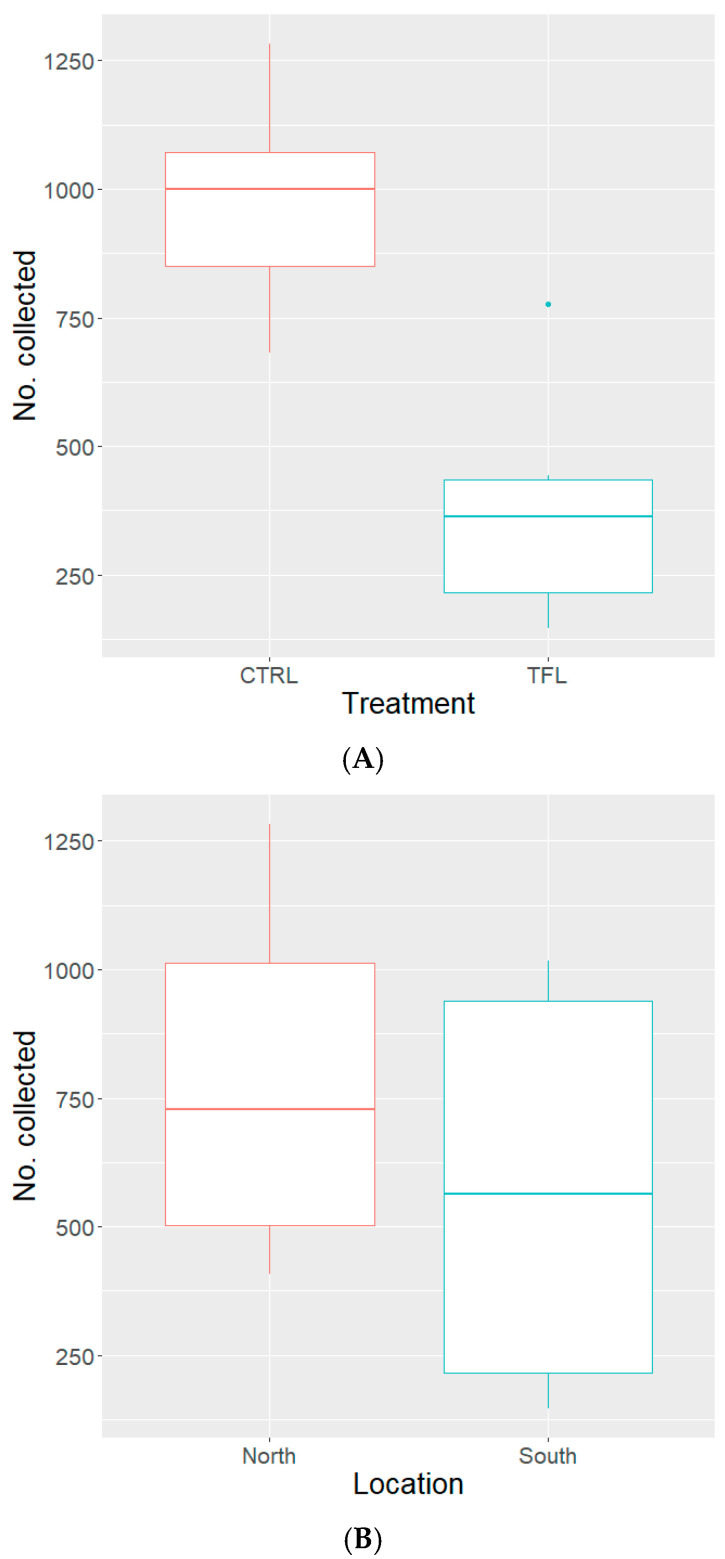
Box and whisker graphs of the median number of ‘WHF’ house flies, representing the “box” (i.e., the interquartile range [IQR]), and variability outside the IQR (i.e., the “whiskers” [Q1/Q3 −/+ 1.5 * IQR]) collected from the baited passive traps protected by treated cotton sachets with transfluthrin (1 g total AI) or water (control) across 6–24 h periods, separated by treatment (**A**) and location (**B**); CTRL = control, TFL = transfluthrin.

Overall, there was a fly capture percentage of 29% to 60%, with a capture average of 45% (*n* = 1350) out of 3000 flies released per replicate. The number of house flies collected from traps protected by the transfluthrin sachets ranged from 146 to 776, with a median of 363.5 (79.6 SE-median) collected over six replicates, and a total of 2274 flies collected from protected traps (transfluthrin) and 5860 from control traps. The number of house flies collected from traps protected by the water control sachets ranged from 681 to 1283, with a median of 999.5 (86.3 SE-median) collected over six replicates. Pooled and averaged ‘wild’ house fly collections by treatment and location are illustrated in Figure 2.

The number of house flies collected by treatment was analyzed via a GLM with a log link function. Model treatment variables were compared to one another through Akaike’s Information Criteria (AIC) and the model with the lowest AIC was selected. The model selected returned an AIC of 163.91 (#collected~treatment * location), while the other models not selected returned an AIC of 171.09 (#collected~treatment) or 180.49 (#collected~location). The number of flies collected in the transfluthrin treatment was significantly different and lower than that of the control, as described in Table 2. Additionally, the locations were analyzed within the same model, via negative binomial GLM with a log link function, and the number of house flies collected from the north or south locations showed no significant difference (Table 2). Furthermore, the interaction between treatment and location were included in the model and found to be significant (Table 2).

### 3.3. Semi-Field with “CAR21” Permethrin Susceptible Strain

**Figure 3:**
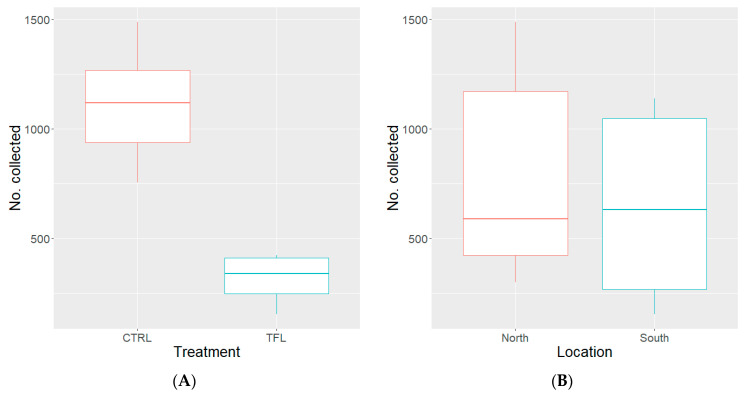
Box and whisker graphs of the median number of ‘CAR21’ house flies (i.e., susceptible strain) representing the “box” (i.e., the interquartile range [IQR]) and variability outside the IQR (i.e., the “whiskers” [Q1/Q3 −/+ 1.5 * IQR]) collected from the baited passive traps protected by treated cotton sachets (*n* = 8) with transfluthrin (1 g total AI) or water (control) across 6–24 h replicates, separated by treatment (**A**) and location (**B**); CTRL = control, TFL = transfluthrin.

Overall, there was a fly capture percentage of 38% to 55%, with an average of 48% (*n* = 1440) out of 3000 flies released per replicate. The number of house flies collected from the traps protected by transfluthrin sachets ranged from 153 to 423, with the median number of 339 (50.5 SE-median) collected over six replicates. A total of 1905 flies were collected from the protected (transfluthrin) traps, while 6669 flies were collected from control traps. The number of house flies collected from traps with water control sachets ranged from 753 to 1487, with the median number of 1119 (128.6 SE-median) collected over six replicates. Pooled and averaged ‘CAR21’ house fly collections by treatment and location are illustrated in Figure 3.

The number of house flies collected by treatment was analyzed via a GLM with a log link function. Model treatment variables were compared to one another through AIC and the model with the second lowest AIC was selected. The model selected returned an AIC of 163.42 (#collected~treatment * location), meanwhile the other models not selected returned an AIC of 163.07 (#collected~treatment) or 183.51 (#collected~location). The second lowest AIC model was selected because the difference between the lowest and the second lowest AIC was 0.35, which is a negligible difference, but would allow comparison of strains to one another, given that they utilized the same treatment variables. The number of flies collected in the transfluthrin treatment was significantly lower than that of the control (Table 2). Additionally, the number of ‘CAR21’ house flies collected from the north or south locations did not differ significantly (Table 2). Furthermore, the interaction between treatment and location was not significant (Table 2).

## 4. Discussion

Here, we were able to describe the resistance of the WHF strain of *M. domestica* located at CMAVE to permethrin compared to the susceptible CAR21 strain of *M. domestica* using a topical bioassay. The resistant population (WHF) was 149.6 times more resistant to permethrin at the LD_50_ value, and 328.2 times more resistant at the LD_90_ value than the susceptible strain (CAR21) (Table 1). Moreover, the populations were screened to identify the molecular mechanism of their resistance, and results showed that the WHF strain carries the *kdr-his* mutation [16]. A ‘*kdr*’ mutation means that the organism carries with it a molecular alteration of the *Vssc* gene to resist the mortality and ‘knockdown’ effect produced by exposure to pyrethroid insecticides [17]. This resistance level coupled with the molecular mechanism of resistance does not match the results documented by Sun et al. [17], where the *kdr-his* strain (NChis) was only 7.1 × more resistant to permethrin than the susceptible strain (aabys). It is possible that the CAR21 strain is simply more susceptible to permethrin than the background strain, aabys, utilized in the previous study highlighted by Sun et al. [17]. Differences in the reporting of resistance ratios of the given resistant strain in question can occur due to comparison to a particular benchmark susceptible strain, as the LD_50_/LD_90_ values for each susceptible strain may differ somewhat [18]. It is also possible that this relatively high resistance ratio is due to other resistance mechanisms that exist in addition to the *kdr-his* mutation. Diverse resistance mechanisms beyond sodium channel modifications (e.g., increased detoxification of pyrethroid, differences in penetration) have been documented in wild fly strains [19,20,21]. Further work should be performed to characterize the physiological underpinnings of this resistance in the WHF strain.

We assessed the use of a short-chain multi-halogenated benzyl pyrethroid spatial repellent (transfluthrin) in protecting a point (i.e., a baited trap) when exposed to released populations of the WHF or CAR21 strains of *M. domestica* in an outdoor semi-field environment between June and August 2023. The results demonstrated that the transfluthrin-protected baited traps collected significantly fewer *M. domestica* than the control (i.e., water) traps (Table 2, Figure 2A and Figure 3A) for both strains. Although transfluthrin successfully reduced capture rates for both strains, the percentage reduction in capture by transfluthrin was slightly lower in the WHF (resistant) strain compared to the CAR21 (susceptible) strain. It is possible that in strains with higher resistance ratios, transfluthrin may be less effective. Furthermore, the protection effect was only assessed for 24 h periods, and more research should be conducted on the longevity of the repellent effect that transfluthrin has against *M. domestica*. In this project, we were attempting to assess the practicality of transfluthrin in protecting a point from fly incursion.

Additionally, there was a discrepancy between collection locations and the strains, where more flies of the WHF strain (Figure 2) were captured in the north trap location than the south trap location than the CAR21 strain (Figure 3). We speculate that this discrepancy between strains may have been an artifact of more efficient shade seeking behavior from the WHF, which hypothetically have a more robust set of behaviors expressed in the field, which is commonly lost through genetic bottlenecking due to colonization [22].

As mentioned in the methods section, we identified a concentration of transfluthrin to apply to the traps and afford a measure of protection to them. Our reliance on the application of 1 g of AI of transfluthrin divided among eight sachets of material was to allow a continuity and comparison of previously documented effects from pest and vector Diptera (*Culex tarsalis* Coquillett and Tabanid flies) exposed to the same concentration [7,15].

Relatively few published studies have assessed transfluthrin against *M. domestica*. However, our results demonstrating transfluthrin as an effective spatial repellent are supportive of the known literature on this topic. For example, Scrivener et al. [8] demonstrated a 81.4% and 79.0% reduction in landing rates of the mixed wild *M. domestica* and *M. vetustissima* Walker within 1 m of the combined transfluthrin- and permethrin-treated glazed tiles and plywood, respectively. Similarly, permethrin-treated scarves reduced the exposure of fly-eye contact by 35% in a study conducted by Robinson et al. [23]. These examples demonstrate that volatile pyrethroids can be used to protect points from *M. domestica*, by limiting their exposure in protected areas.

Recently, given the resistance of *M. domestica* to commonly used synthetic chemical controls like permethrin, a greater emphasis has been placed on exploring natural products, such as parasitoids, fungi, and plant-derived essential oils, as spatial repellents. There have been promising results in the literature demonstrating the use of plant essential oils as spatial repellents. For example, Khater and Geden [24] demonstrated the repellency of several essential oils against protein-starved adult *M. domestica*, specifically vanillin, p-menthane-3,8-diol (PMD), and Neem oil. An additional example is seen by blending essential oils, as demonstrated by Hazarika et al. [25] who combined clove oil, citronella oil, lemon grass oil, and camphor into an evaporative tablet; generating repellency (>81%) against M. domestica for up to 8 days. Similarly, plant essential oils and synthetic pyrethroid spatial repellents could be combined to create a synergized product against *M. domestica* or other noxious pests, comparable to Andreazza et al. [26] who combined transfluthrin with six plant-derived mosquito repellents (geranyl acetate, (E)-β-farnesene, (−)-borneol, (±)-citronellal, camphor, and eucalyptol). The results were promising and demonstrated significantly higher repellency against *Aedes aegypti* (L.) than the plant-derived repellents or transfluthrin alone.

## 5. Conclusions

In conclusion, these results demonstrate a pattern of efficacy of the use of transfluthrin in protecting certain points in areas such as animal/food processing facilities, open wounds on animals, or open food sources. Multiple opportunities for research are available based on these results supporting the use of synthetic pyrethroids, such as transfluthrin in repelling difficult to control pests and vectors like *M. domestica*. Additionally, as observed by Andreazza et al. [26], there is potential in synergizing this synthetic spatial repellent with plant essential oils in the future to enhance their protective properties against *M. domestica* as well as other filth flies.

## Figures and Tables

**Table 1:** Relative susceptibility of the two *Musca domestica* fly strains, resistant WHF and susceptible CAR21, to permethrin topical bioassay.

Fly Strain	*N*	Slope (SE)	LD_50_ (95% CL) (ng/fly)	LD_90_ (95% CL) (ng/fly)	Resistance Ratio LD_50_ (RR)	Resistance Ratio LD_90_ (RR)
CAR21	400	1.56 (0.13)	11 (10–13)	26 (22–32)	--	--
WHF	400	0.80 (0.08)	1720 (1149–2073)	8534 (6134–13,642)	149.6	328.2

**Table 2:** Negative binomial generalized linear model with log link results assessing released ‘WHF’ and ‘CAR21’ colonies of house flies collected in a semi-field enclosure from the Captivator traps protected by treated cotton sachets (*n* = 8) with transfluthrin (1 g total AI) or water (control).

Response Var.	Treatment	Param. Est.	S.E.	Z	*p*
Collected ‘WHF’ house flies	Intercept	6.93	0.15	46.47	<0.0001
	Treatment	−0.63	0.21	−2.98	0.003
	Location	−0.09	0.21	−0.41	0.69
	Treatment × Location	−0.84	0.30	−2.78	0.005
Collected ‘CAR21’ house flies	Intercept	7.08	0.14	49.50	<0.0001
	Treatment	−1.13	0.20	−5.56	<0.0001
	Location	−0.13	0.20	−0.63	0.53
	Treatment × Location	−0.28	0.29	−0.96	0.337

## Data Availability

The data presented in this study are available on request from the corresponding author (as of January 2024).
